# Novel Uses and Challenges of Artificial Intelligence in Diagnosing and Managing Eyes with High Myopia and Pathologic Myopia

**DOI:** 10.3390/diagnostics12051210

**Published:** 2022-05-12

**Authors:** Ran Du, Kyoko Ohno-Matsui

**Affiliations:** Department of Ophthalmology and Visual Science, Tokyo Medical and Dental University, Tokyo 113-8510, Japan; duran.tmdu@gmail.com

**Keywords:** diagnosis and management, high myopia, pathologic myopia, artificial intelligence, machine learning, deep learning

## Abstract

Myopia is a global health issue, and the prevalence of high myopia has increased significantly in the past five to six decades. The high incidence of myopia and its vision-threatening course emphasize the need for automated methods to screen for high myopia and its serious form, named pathologic myopia (PM). Artificial intelligence (AI)-based applications have been extensively applied in medicine, and these applications have focused on analyzing ophthalmic images to diagnose the disease and to determine prognosis from these images. However, unlike diseases that mainly show pathologic changes in the fundus, high myopia and PM generate even more data because both the ophthalmic information and morphological changes in the retina and choroid need to be analyzed. In this review, we present how AI techniques have been used to diagnose and manage high myopia, PM, and other ocular diseases and discuss the current capacity of AI in assisting in preventing high myopia.

## 1. Introduction

Myopia is a global health issue, and the prevalence of myopia has increased significantly in the past five to six decades [1]. In urban areas of China, Taiwan, Hong Kong, Japan, Singapore, and South Korea [2,3,4,5,6,7], 80–90% of high school students are myopic and 10–20% of them have high myopia [1,8]. The same prevalence has been observed in North America, Germany, Spain, and Russia [9,10,11,12]. The worldwide increase in the prevalence of myopia and PM indicates that myopia-related blindness will increase worldwide in the future [13,14,15,16,17,18,19,20,21,22,23,24,25,26,27,28,29]. A lack or shortage of myopia specialists is a great concern to governmental leaders, and the control of myopia has been a national policy in China [30].

In PM eyes, there is an increase in the axial length and the presence of a posterior staphyloma, a deformity of the posterior segment of the eye [31,32,33,34,35]. Following a deformation of the sclera, the neural retina is mechanically damaged and blinding pathologic changes develop in the macular region. The eyes are then said to have myopic maculopathy, which is the main sight-threatening complication. In addition, it has been reported that the cost for one myopic patient would be over seven hundred United States dollars/year and 17 thousand United States dollars during the patient’s lifetime in Singapore [36]. In China, it is estimated that myopia-associated productivity loss is about 244 billion United States dollars/year [30,37]. These values indicate that myopia is an increasingly serious public health problem with a high economic burden. Because myopic maculopathy is generally progressive and irreversible, interventions to prevent the progression of myopic eyes to PM, continuous surveillance, and slowing the progression of PM are highly recommended. However, the number of well-trained myopia specialists is insufficient worldwide and the diagnosis of myopic maculopathy is difficult for general eye care providers, e.g., optometrists or general ophthalmologists, and a continuous monitoring of every myopic patient is inefficient in both time and cost. For example, various lesions of myopic maculopathy often co-exist in the same eye, which makes their appearance difficult to interpret. Thus, there is a great need for automated methods that can be used in a cost-efficient way to assist physicians in monitoring PM and to manage PM patients who need the care of specialists.

Artificial intelligence (AI) has been identified as one of the key drivers of the Fourth Industrial Revolution [38]. Because of the growth of digital databases, the number of AI-based applications in the medical field based on Python or C has increased immensely in recent years [39,40]. One of the main parts of AI is machine learning (ML), which not only has a powerful capacity for statistical analyses but also has a great ability to manipulate data and perform complex operations to find relationships among the many biological characteristics. As an evolutionary form of ML, deep learning (DL) enhances these advantages and has reached a new high by processing data through information in hidden layers.

**Figure 1:**
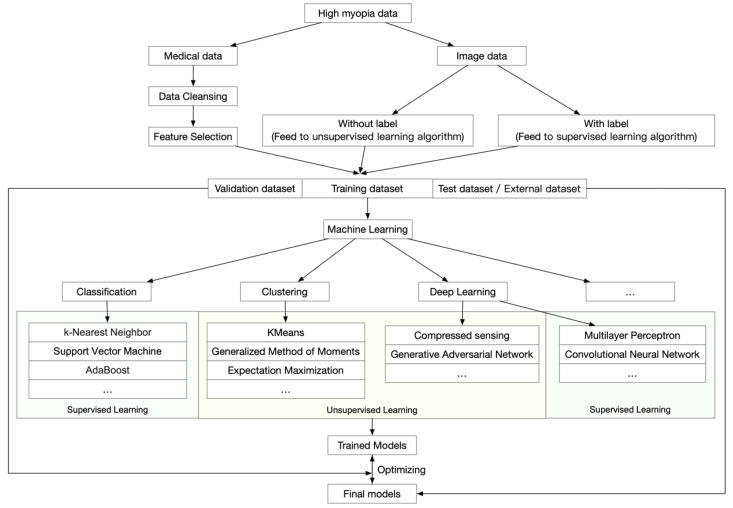
General workflow of artificial intelligence analyses of high myopia and pathologic myopia.

Many successful models and platforms have been established for screening and diagnosing age-related macular degeneration [41,42,43], diabetic retinopathy [44,45], and glaucoma [46]. These applications focused on analyzing ophthalmic images to diagnose the disease and to determine prognosis from these images. However, in addition to a general workflow, which is shown in Figure 1, high myopia and PM generate even more data because both the ophthalmic information and morphological changes of the retina and choroid need to be analyzed.

In this review, we examine how AI has been applied for the diagnosis and management of high myopia and PM.

## 2. Data-Driven AI in High Myopia and Pathologic Myopia

PM is associated with an elongation of the axial length of the eye, which is usually associated with morphological changes in the sclera, choroid, Bruch’s membrane, retinal pigment epithelium, and neural retina. In addition, due to increases in the progressive and excessive axial lengths, highly myopic eyes also have high refractive errors and related ophthalmic changes. These changes may be further amplified when the eye undergoes refractive or cataract surgery due to the excessive length of the eye. Thus, it is expected that high myopia will generate a considerable amount of data during a long-term follow-up period, which would require an efficient method to analyze and interpret the findings.

Earlier, redundant and inconsistent data were collected due to the non-integrated and fragmented data management procedures. This has led to information quality problems, which has hampered the acquisition of an accurate diagnosis, resulting in poor management of myopic eyes.

With the recent creation and general distribution of digital hospital information systems, an opportunity has opened up for determining the onset and progression of PM through a much larger set of data. This has advanced our understanding of PM with more comprehensive perspectives and on more solid theoretical bases.

Data-driven AI studies are usually performed using ML techniques because they can detect different categories, obtain information buried in a large amount of data, and optimize the model that best fits the data. The models that are regressed by training data would verify the capacity for data categorization. ML techniques involve supervised learning, semi-supervised learning, and unsupervised learning. They include many methods such as kernel ridge regression, support vector machines (SVM), nearest neighbors, gaussian processes, naive Bayes, random forests, neural networks, and others. Further evolutional methods such as extreme gradient boosting (XGBoost) and light gradient boosting machine (LightGBM) supply more chances in regression models and can determine potential relationships to understand the occurrence and progression of high and pathologic myopia. With these powerful methods, representative patterns can be statistically calculated and extracted for ensemble predictive models.

Earlier studies reported that the incidence of myopia had reached 84.6% in elementary school children and 95.5% in university students in China [47,48,49], and it is not difficult to believe that such levels are not unique to China. Thus, it is urgent to monitor eyes with high myopia at an earlier stage, which raises the need for AI-assisted screening techniques. In areas with high levels of myopia, several data-driven studies on high myopia have reported that DL learning models can be used to solve real problems with sensible solutions (Table 1). The ML models have shown that the refractive errors and the risk of high myopia (myopia ≤ −6.0 diopters) that develop within ten years are predictable in school-aged children [50]. In this approach, the random forest model, generalized estimating equation model, and mixed-effects model were fitted and evaluated by the coefficient of determination (R2), the root mean square error (RMSE), mean absolute error (MAE), and characteristics of the area under the receiver operating curves (AUC). The model was tested by both internal and external datasets. Typically, the random forest model had the best performance and the AUC reached as high as 0.802 to 0.976. This approach provided evidence for transforming clinical practice, health policy-making, and precise individualized interventions regarding the practical control of school-aged myopia by employing big data and ML. However, in some circumstances where the clinical data are not available, it may be difficult for physicians to manage high myopia patients. To address this, ML models were also designed and trained to play roles in analyzing eyes with high myopia. By training with the wavefront aberrometry values through the XGBoost algorithm, DL models have been used to predict the subjective refractive errors, and the mean absolute error between true values and predicted values ranged from 0.094 to 0.301 diopters, and the combination of machine learning and aberrometry based on wavefront decomposition basis will aid in the development of refined algorithms [51]. Furthermore, highly myopic eyes often have hyperopic refractive errors after cataract surgery, despite the use of partial coherence interferometry, which could eliminate biometric errors. Through XGBoost regression, AI models trained by medical records extracted from myopia patients could improve the accuracy of implementing IOL power in high myopia with cataracts [52].

In addition, in situations where only limited information can be accessed, electrooculographic (EOG) data could also be used to train ML models in classifying myopic refractive disorders. It has been reported that when the logistic regression model, Naïve Bayes model, and random forest model were trained by EOG data, the random forest model had the best performance with a sensitivity of 95.5% and a specificity of 96%. The total classification accuracy reached 90.91%, and the achieved models could inspire novel approaches to clinical screening of myopia when general data are not available [53]. Furthermore, because the axial length value is a key indicator for high myopia, simply assessing the change in axial length can be used to evaluate the myopia progression. More specifically, these methods can be used by practitioners to judge the true extent of myopia progression before performing a cycloplegic refraction examination. Linear regression, SVM, and bagged trees have been used to predict increases in axial length in adolescents. From an evaluation of the performance of models by five-folded cross-validation, the linear model achieved a high level of precision with an R square value of 0.87 [54].

In addition to these methods of predicting the actual outputs, there are other ways to use AI algorithms. It is generally accepted that clinical data tend to be imperfect and may lack different parts during clinical research because each performed examination is required to test the evidence-based hypothesis. However, these imperfect data would be a high barrier for research and the understanding of these disease processes. One of the benefits of ML algorithms is that they can fill in the missing values based on a scientific method, and the results can be closer to the true value. This will lead to a better understanding of the occurrences and progression of the disease processes. Furthermore, even with abundant data or features that can be assessed, physicians still need to determine how to filter out important values to test a hypothesis. In addition to traditional methods such as the principal component analysis (PCA), ML algorithms supply multiple choices for data dimension reduction, such as randomized singular value decomposition-based PCA, spectral embedding, isomap embedding, and others. These algorithms offer opportunities for clinicians to analyze the abundant data and to determine ways to test their hypotheses.

For myopia control, it is widely known that the environment, especially luminance and ultraviolet, plays important roles in affecting the progression of myopia. As the nature of collecting monitoring environmental data is complex, it is difficult to implement monitoring widely in the public. Through luminance, ultraviolet light levels, and step number data, AI models could be trained in different indoor and outdoor locations. These methods can be useful monitoring tools for community- or school-based public health interventions or individual health management [55].

ML models have been typically used to fill in missing clinical data and to select features that were highly correlated with the myopia in adolescents [56]. Features selected by ML learning algorithms have been used to explore the potential risk factors that affect the severe axial length elongation in highly myopic eyes. These approaches are particularly important because they provide reference data for physicians when faced with complex situations. To screen for high myopia in rural areas where myopia specialists or essential instruments are not available, these predictive values would be important indicators for high myopia screening and for monitoring the progression of myopia.

## 3. Image Driven AI in High Myopia and Pathologic Myopia

Eyes with PM have a high degree of myopia with degenerative changes in the retina and choroid, especially in the posterior pole. Because of the different image contents, the method and purpose of the assessments are different. The most commonly used AI technique in highly myopic eyes is the assessment of fundus photographs and optical coherence tomography (OCT) images. Both are noninvasive and can be recorded frequently without any side effects.

**Figure 2:**
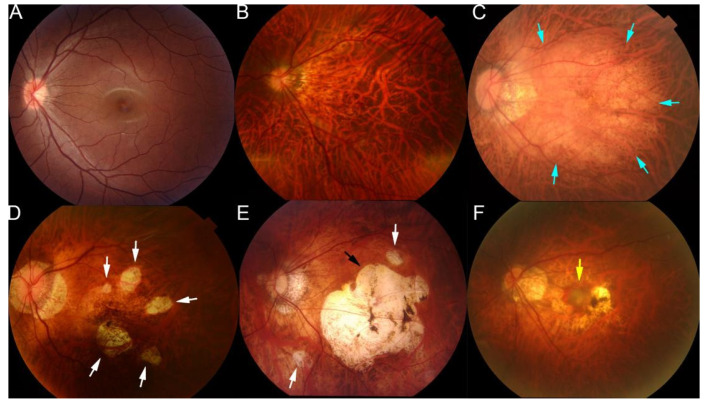
Representative fundus photographs showing the different types of lesions of maculopathy in eyes with pathologic myopia. (**A**) Normal fundus image. (**B**) Tessellated fundus. (**C**) Diffuse atrophy around optic disc and posterior fundus (blue arrows). (**D**) Patchy atrophy fundus (white arrows). (**E**) A fundus image from a left eye with macular atrophy at the center of posterior fundus (black arrow). Patchy atrophy (white arrow) as well as diffuse atrophy background can also be seen. (**F**) Fundus image with myopic choroidal neovascularization at the center of fundus (yellow arrow). Reprinted from *Deep Learning Approach for Automated Detection of Myopic Maculopathy and Pathologic Myopia in Fundus Images*, Vol 5, Pages No. 1235–1244, Copyright (2021), with permission from Elsevier.

The lesions of myopic maculopathy have been classified according to the META-PM study group classification [58,59,60]. From a review of earlier studies and classifications, an international panel of myopia researchers proposed a simplified system for PM called the META-PM system, and lesions of myopic maculopathy were classified into five categories based on color fundus photographs: category 0 represents “no myopic retinal lesions”; category 1 represents “tessellated fundus only”, which can be observed with choroidal vessels clearly around fovea as well as arcade vessels; category 2 represents “diffuse chorioretinal atrophy”, which is a yellowish atrophy lesion that usually starts around the optic disc and gradually enlarges to the macula area; category 3 represents “patchy chorioretinal atrophy”, which a grayish-white atrophy with a clear margin; and category 4 represents “macular atrophy”, which is a well-defined round atrophic lesion with a grayish-white color that covers the macular area [58]. In addition, the plus lesions, e.g., lacquer cracks, choroidal neovascularization, and Fuch’s spot, could also be confirmed in the fundus images in typical cases. These PM-related fundus changes were mainly used as anchors in training fundus image-based AI models (Figure 2).

**Figure 3:**
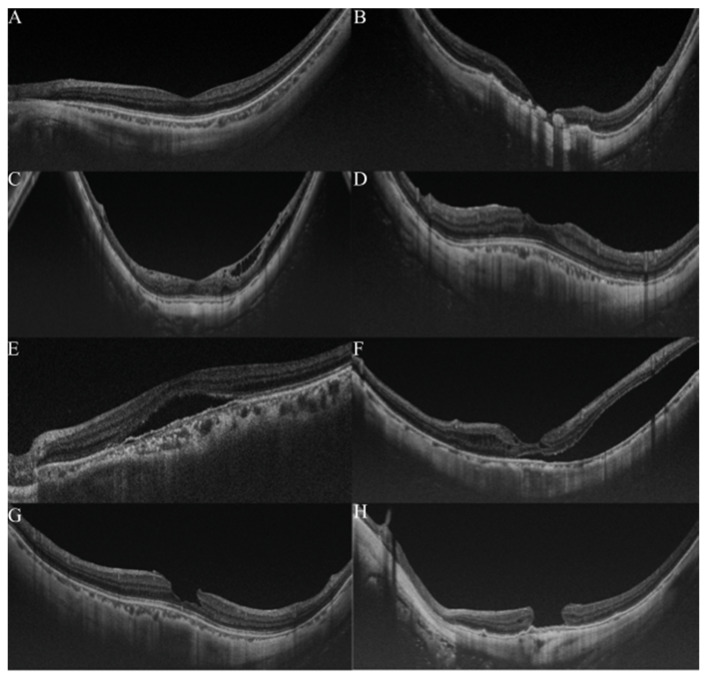
Grading samples of myopic maculopathy in ocular coherence tomographic (OCT) images. (**A**). Myopic eye without myopic maculopathy. Each of retinochoroidal layer is clearly seen. (**B**). Myopic neovascularization (MNV). Hyperreflective materials can be seen above the retina pigment epithelium (RPE), and this component is attenuated in the tissue coherence signals below. (**C**). Retinoschisis. The splitting of the inner retina from the outer retinal layers with multiple perpendicularly aligned columnar structures connecting the split retinal layers. (**D**). Dome-shaped macular (DSM). An inward bulging of the retina pigment epithelium above the baseline connecting the RPE lines on both sides away from the DSM. (**E**,**F**). Retinal detachment. The neurosensory retina is detached from the RPE. (**G**,**H**) Macular hole. A tear above the RPE layer and an anvil-shaped deformity of the cracked edges of the retina. Reprinted from *Validation of Soft Labels in Developing Deep Learning Algorithms for Detecting Lesions of Myopic Maculopathy from Optical Coherence Tomographic Images*, Copyright (2021), with permission from Wolters Kluwer Health.

OCT is widely used for detecting and analyzing retinochoroidal disorders, and it has become the general method of examination used to assess the retina of myopic patients. Typically, in pathologic myopia, OCT images were always used to observe the pathological retinochoroidal changes or progression of retina degeneration (Figure 3). The OCT images were mainly used to examine myopic traction maculopathy (MTM), which is a spectrum of foveal tractional changes in highly myopic eyes [61]. All MTM-related alterations, such as retinoschisis, retinal detachment, and macular holes, can be identified in OCT images. Thus, it is expected that AI could assist physicians in grading pathologic myopia retinochoroidal changes in OCT images.

According to the typical changes shown Figure 2 and Figure 3, it would be efficient and useful once trained models play roles in recognizing these lesions from different modalities. However, photographs of the fundus of the eye contain redundant color information and the various appearances of the myopic lesions make it difficult for ML methods to extract pathogenic patterns that could be used for categorizing the causative disease more accurately. Furthermore, because multi-lesions or co-existing lesions were generally found in highly myopic eyes, redundant information provides a higher barrier for fitting ML models.

**Figure 4:**
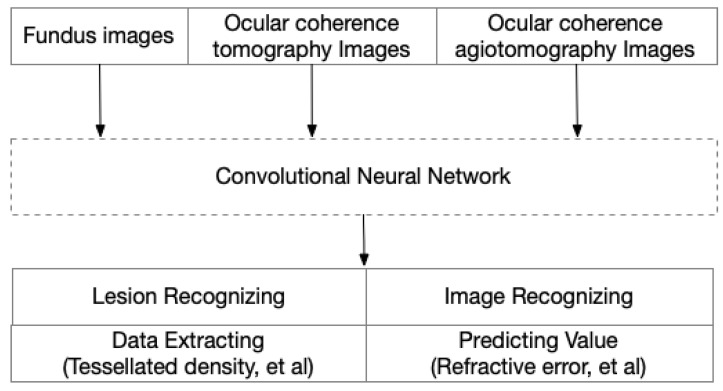
Image-driven artificial intelligence in high myopia and pathologic myopia.

Deep learning (DL) is a sub-type of ML that uses additional hidden layers to manage more complicated nonlinear patterns in the data. The rapid development of the software library for ML and the many deep convolutional neural networks (CNN), such as AlexNet, VGGNet, ResNet, Inception, DenseNet, and EfficientNet, has greatly increased the accuracy in complicated medical images. Recently, CNN was used to train models with a carefully balanced network between width and depth, which supported a high resolution with greater accuracy and efficiency. These advantages made it possible to analyze complex images in PM eyes in an automated way. Furthermore, with this redundant information, DL models could also extract information for predicting values, even though these data always have less readability (Figure 4).

Many image-based DL models are ready to be used in the clinical management of PM (Table 2), and these models were mainly implemented by Python or C. In addition to the basic information that can be obtained from fundus images, such as the status of the posterior retina, additional information can be detected by AI models. To diagnose high myopia correctly and automatically, after being trained by fundus images and validated by an external dataset, deep learning models can predict a high myopia fundus with an AUC of 0.9968 for the recognition of low-risk high myopia and 0.9964 for the recognition of high-risk high myopia [62]. Furthermore, the refractive errors can also be extracted through fundus images. Trained by fundus images through ResNet, new information such as the refractive error, the spherical and cylindrical components, and the mean absolute error (MAE) between true values and predicted values were minimized to 0.56 diopters for estimating the spherical equivalent refractive error [63].

Because the degree of myopic maculopathy can be used to follow the progression of PM [64], early detections of the lesions of myopic maculopathy accompanied by active follow-up and prompt treatment of complications are important in protecting patients with high myopia from permanent vision loss. In grading the severity of the PM, trained DL models and assembled systems of these models can achieve high sensitivity and specificity in identifying the different types of lesions of myopic maculopathy. Through training by a large number of fundus images from pathologic myopia eyes, models could achieve accuracies of around 85.7–99.4% in recognizing diffuse atrophy, patchy atrophy, macular atrophy, and myopic choroid neovascularization. These findings have promise for facilitating clinical diagnosis and healthcare screening for PM on a large scale [65,66,67,68]. Because tessellation might be the first sign that a highly myopic eye will become pathologic [69], the screening for tessellated changes in myopic fundus is critical for monitoring the progression of high myopia. It is feasible and efficacious to extract quantitative information about the density of the tessellation from fundus images by DL-based image processing [70]. In addition to the successfully extracted information from fundus images, it is feasible and efficient to be used in population screening as a new quantitative biomarker for the thickness of the subfoveal choroid, which would lead to further investigations for pathological myopia and lower visual acuity.

DL algorithms can achieve more possibilities in analyzing retinochoroidal changes present in OCT images. For example, DL models were able to classify OCT images with myopic macular lesions and the type of myopic macular lesions with high accuracy [71,72,73]. Furthermore, for severe high myopic eyes, the image quality in myopic eyes is not necessarily good due to the long axial length, which can lead to an ambiguous or uncertain diagnosis. DL models can also be trained based on answers given by different specialists and can then predict the possibilities of a risk for a specific MTM lesion. This approach would be especially helpful in complicated cases and further inspires novel use for DL models [75]. Furthermore, because choroidal thinning is recognized as being highly correlated with the progression of high myopia, it would be critical if AI could assist in segmenting the choroidal layers automatically. An earlier study reported that, in swept-source OCT images, trained AI models could resemble the macular focused scans and that the segmenting choroidal volume has an accuracy of intersection over union of 0.92 [74]. Another study also reported that trained DL models could segment and quantify choroid in OCT images with excellent performance. The mean dice coefficient between the region segmented by automatic and manual methods was 93.87 ± 2.89% [76].

These trained DL models and DL-related approaches suggest the possibility of conducting highly accurate screening of ocular diseases using AI. It is possible that automatic manners would assist physicians and further reduce the workload for ophthalmologists. In addition, high myopia patients living where a lack of myopia specialists is present would also benefit from these automatic manners and may help prevent blindness by timely screening and referral.

## 4. Challenges

With the immense increase in the amount of digital data, researchers or institutes could fulfill the basic conditions for data extraction and model construction. Ophthalmic images as well as follow-up data have all been used for extracting specific features and for furthering train AI models. Currently, most AI-assisted diagnoses mainly focused on high myopia status predictions and PM-related lesion recognition, especially for different PM lesions. According to the literatures above, it is obvious that not only fundus images or OCT images but also abundant medical data could be used for training AI-assisted models in solving specific questions. It is expected that AI would play roles in assisting in diagnosing lesions or in accelerating the progression of clinical research in the foreseeable future. However, some challenges still remain and need to be further handled for the current situation.

The marked escalation of myopia and PM makes it essential to develop and apply AI models for the diagnosis and management of PM even though it is not an easy task. For monitoring the progression of high myopia in adolescents, data bias may continue to be present among school-aged children due to the unequal distribution of educational resources and geographical conditions. These issues may lead to feature bias among the children enrolled such as the proportion of near work and outdoor pursuits, lifestyle habits, genetic differences, and other factors. These biases in the data may have a profound impact on the generalization of trained AI models, and the performance of cross-region models may be restricted. Even though many models are available in predicting the presence of high myopia-related features in myopic adults, the predictive prognostication for PM-induced optic morphological changes has not been fully carried out because of the diagnostic challenges in adulthood. In highly myopic eyes, the morphological changes in the intrapapillary and parapapillary region have not been fully determined, especially regarding the status of the laminar cribrosa and tissues around the optic disc region in highly myopic eyes. A better understanding of the peripapillary changes would be helpful for further AI-assisted diagnoses in screening and monitoring PM-related sight-threatening complications.

On the other hand, the data within the images may cause some difficulties in performing automated analyses. First, accurate training and validation of AI systems across multiethnic data sets are required. Because most image-based AI models are based on images from a single health center, it is possible that model performances may not be used across racial boundaries. Second, because long axial lengths are commonly seen in highly myopic eyes, considerable experience and expertise may be necessary to record high quality images from highly myopic eyes. Moreover, it is common to see various pathologies co-existing in PM eyes, and these lesions may lead to ambiguous or uncertain diagnosis of labeling PM lesions and further increase the threshold for training and validating the AI models.

To address these limitations during the implementation of AI-assisted automated analyses in highly myopic eyes, extensive research and substantial expenditures are required. A deeper understanding of high myopia and the PM-related risk factors based on big data are essential. These important variables or relationships could lead to a better performance of automatic techniques and be critically helpful for the screening for PM. Furthermore, multimodal medical image fusion techniques may be of great help in further assistance in AI-assisted PM detection. A combination of two or more fundus images or a combination of fundus and OCT images may improve the image content and directly preserve information for physicians or the training of AI models [78].

Finally, as each AI model was trained with specific dataset or data type, the performance of models may be restricted to solving only questions through datasets with restrictions of the same structure. It is expected that, in the future, the evolution of computing algorithms could make it more generalizable or easier to distribute across various data types. Additionally, as the model should ideally be consistent and generalizable in the clinic even across multiethnic eyes, multicenter cooperation has been imbued with a stronger sense of urgency, especially in nations with heterogeneous populations. Last but not least, since machine learning models were born with overfitting and reached a promising accuracy, currently, it is imageable that future models would make trades between accuracy and floating-point operations per second. Scaling accuracy and efficiency by controlling network depth, width, and image size would makes the distribution of models easier.

## 5. Conclusions

In conclusion, the prevalence of high myopia and PM is rapidly increasing, and they require long-term follow-up monitoring and timely interventions. Meeting with a general situation of unevenly distributed medical resources, future AI-assisted studies should focus more on telemedicine, which could be easily and efficiently distributed in rural areas, which would be of great help to high myopia screening and control in areas that lack myopia specialists. To promote personalized monitoring and treatment of highly myopic eyes, a rational policy of support and a deeper level of cooperation are needed. The relevance and effectiveness of AI in myopia are still in dispute. Before the widespread use of AI healthcare for high myopia, several technological and clinical difficulties must be overcome.

## Figures and Tables

**Table 1:** Data-driven artificial intelligence (AI) models in high myopia and pathologic myopia.

Research	Year	Materials	Participants	AI Methods	Main Outcome	Evolutions and Performance
Lin, H. et al. [50]	2018	Refraction data	School-aged children	ML	Predicting the presence of high myopia	AUC: 0.802–0.976
Kaya, C. et al. [53]	2018	electrooculographic data	Adults (25–65 years old)	ML	Detecting hypermetropia and myopia refractive disorders	Sensitivity: 95.5%; specificity: 96%; classification accuracy: 90.91%
Ye, B. et al. [55]	2019	luminance, ultraviolet light levels, and step number data	Myopia patients	ML	Differentiating indoor and outdoor locations	Accuracy: 0.827–0.996; AUC: 0.90–0.99
Rampat, R. et al. [51]	2020	Wavefront aberrometry data	General population	ML	Predicting subjective refraction	mean absolute error: 0.094–0.301 diopters
Tang, T. et al. [54]	2020	Medical data	School-age myopic children	ML	Estimating physiological elongation of axial length	R square equals 0.87
Wei, L. et al. [52]	2020	Medical data	Myopia patients	ML	Improving the accuracy of IOL power predictions	mean absolute error: 0.25–0.29; median squared errors: 0.06–0.09
Yang, X. et al. [56]	2020	Medical data	Primary school children	ML	Studying influence of related factors on incidence of myopia in adolescents	Accuracy equals 0.92–0.93; Precision equals 0.95; Sensitivity equals 0.94; f1 equals 0.94; AUC equals 0.98; Specificity equals 0.94
Li, S.M. et al. [57]	2022	Medical data	Primary school children	ML	Detecting risk factors for myopia progression	Combined weight: 77%; Accuracy: over 80%

AUC, area under the receiver operating characteristic curves; ML, machine learning.

**Table 2:** Image-driven artificial intelligence (AI) models in high myopia and pathologic myopia.

Title	Materials	Year	Participants	Net Structure	Main Outcome	Evolutions and Performance
Varadarajan, A.V. et al. [63]	Fundus images	2018	Adults (40–69 years old)	ResNet	Extract refractive error	Mean absolute error of 0.56–1.81 diopters
Hemelings, R. et al. [71]	Fundus images	2020	Not Mentioned	UNet++	Detect PM and semantic segmentation of myopia-induced lesions	AUC: 0.9867; Dice score: 0.8001–0.9303 F1 metrics: 0.7059–0.9869
Wan, C. et al. [62]	Fundus images	2021	General population	VGG-Face	Grade the risk of high myopia	AUC: 0.9964–0.9968
Du R. et al. [65]	Fundus images	2021	Adults	Efficient Net	Identify the different types of lesions of myopic maculopathy	Accuracies: 87.53–97.50%; AUC: 0.881–0.982; sensitivity: 0.370–0.872; specificity: 0.945–0.983
Lu, L. et al. [66]	Fundus images	2021	General population	ResNet	Automatically identify pathologic myopia, classify myopic maculopathy, and detect “Plus” lesions	AUC: 0.979–0.995; Accuracies: 0.967–0.994; Sensitivity: 0.684–0.978; Specificity: 0.970–0.995
Shao, L. et al. [70]	Fundus images	2021	Adults (50–93 years old)	ResNetFCN	Quantitatively assess the fundus tessellated density and associated factors	Accuracy: 0.9652; Sensitivity: 0.7247; Specificity: 0.9605
Li, J. et al. [67]	Fundus image	2022	Adults	Dual-stream DCNN	Detect pathologic myopia and tessellated fundus	AUC: 0.970–0.998; Sensitivity: 81.1–98.8%; Specificity: 95.9–99.6%.
Sogawa, T. et al. [72]	OCT images	2020	Adults	Multi-neural network	Identify images with myopic macular lesions and images with myopic macular lesions	AUC: 0.970–1.000; Accuracy: 67.6–96.5%; Sensitivity: 90.6–1.000%; Specificity: 94.2–100%.
Li, Y. et al. [73]	OCT images	2020	Adults	VGGNet	Identify vision-threatening conditions	AUC: 0.961–0.999
Cahyo, D.A.Y. et al. [74]	OCT images	2020	Not Mentioned	Bidirectional C-LSTM U-Net	Volumetric Choroidal Segmentation	AUC: 0.92
Du, R. et al. [75]	OCT images	2021	Adults	DarkNet	Detect myopic neovascularization, myopic traction maculopathy, and dome-shaped macula	AUC: 0.946–0.985; AUPR: 0.653–0.908
Chen, H.J. et al. [76]	OCT images	2022	Adults	Region-based CNN	Segment and quantify of choroid	mean dice coefficient between automatic and manual methods: 93.87% ± 2.89%.
Park, S.J. et al. [68]	OCT images	2022	Adults	Multi-neural network	Detect pathologic myopia	Accuracy: 95%; Sensitivity: 93%; Specificity: 96%, AUROC: 98%
Wu, Z. et al. [77]	Fundus images/OCT images	2022	Adults	Multi-neural network	Predict optical coherence tomography (OCT)-derived high myopia grades based on fundus photographs	AUC: 0.895–0.969; Accuracy: 0.85.43–94.21%

AUC, area under the receiver operating characteristic curves; PM, pathologic myopia; OCT, ocular coherence tomography; DL, deep learning; AUPR, areas under the precision–recall curves.
